# Death of Dr. Thomas Spencer

**Published:** 1857-07

**Authors:** 


### MISCELLANEOUS ITEMS.

*Death of Dr. Thomas Spencer.*

Dr. Thomas Spencer, a distinguished physician of Central New York, who has been residing in this city for several years past, died at his residence here on Saturday last. He occupied a professorial chair in the medical college of Geneva for fifteen years; afterwards filled a chair in the medical college of Chicago, and five years ago came to a professorship in the Philadelphia College of Medicine. In medicine, politics, or authorship he was equally at home. He officiated as surgeon in the American army in Mexico during the war, was a member of the New York Legislature, and the author of a work on the "Chemistry of Animal Life," in which some original and striking views are presented. A number of elaborate articles from his pen will be found in the early volumes of this Journal. —*Phil. Med. and Surg. Journal.*

